# A Typical but Underdiagnosed Nasal Cavity Mass

**DOI:** 10.5334/jbsr.1366

**Published:** 2018-04-19

**Authors:** Stephanie Vanden Bossche, Geert De Vos, Marc Lemmerling

**Affiliations:** 1 Department of Radiology, AZ Jan Palfijn, Ghent, BE; 2 Department of Otorhinolaryngology, AZ Sint-Lucas, Ghent, BE; 3 Department of Radiology, AZ Sint-Lucas, Ghent, BE

**Keywords:** REAH, respiratory epithelial adenomatoid hamartoma, olfactory cleft, nasal cavity mass

## Abstract

Respiratory epithelial adenomatoid hamartoma is a relatively new diagnosis, only added to the World Health Organization classification of tumours in 2005. The lesion results from non-neoplastic overgrowth of glandular tissue in the nasal cavity and rarely in de sinus cavities and is often associated with nasal polyposis. The classical appearance of a bilateral mass in the olfactory cleft causing widening of the olfactory cleft allows the radiologist to suggest the diagnosis on computed tomography or magnetic resonance imaging.

## Introduction

The term *respiratory epithelial adenomatoid hamartoma* (REAH) was introduced by Wenig and Heffner in 1995 to define the lesions they found in the nose and sinus cavities in their series of 31 patients. They described it as “a proliferation of glands lined by multi-layered ciliated respiratory epithelium, often with admixed mucocytes, arising in direct continuity with the surface epithelium, which invaginated downward into the submucosa” [1]. Since the entity is relatively unknown among radiologists, otorhinolaryngologists and pathologists, it is often underdiagnosed. A correct preoperative diagnosis is important because this strictly benign entity requires only minimal invasive surgery. We present a typical example of bilateral REAHs originating from the olfactory clefts in a 49-year-old woman.

## Case Report

**Figure 1:**
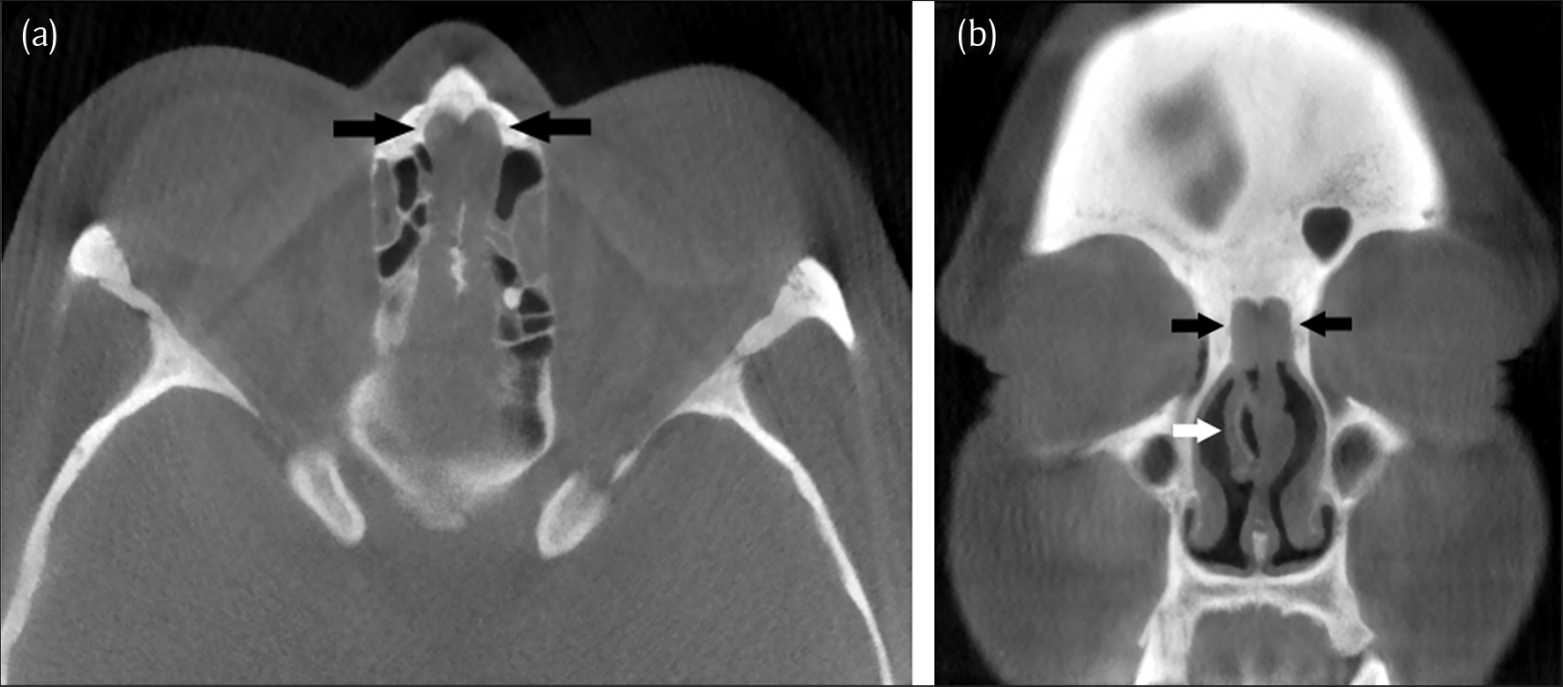
Axial **(a)** and coronal **(b)** CT images in bone window settings demonstrate the typical imaging findings. Bilateral opacification of the olfactory cleft leading to a total width of 1 cm (distance between the black arrows). The sharply delineated soft-tissue mass (black arrows) originate anteriorly and superiorly from the middle concha (white arrow). There is bone remodelling but no bone erosion.

**Figure 2:**
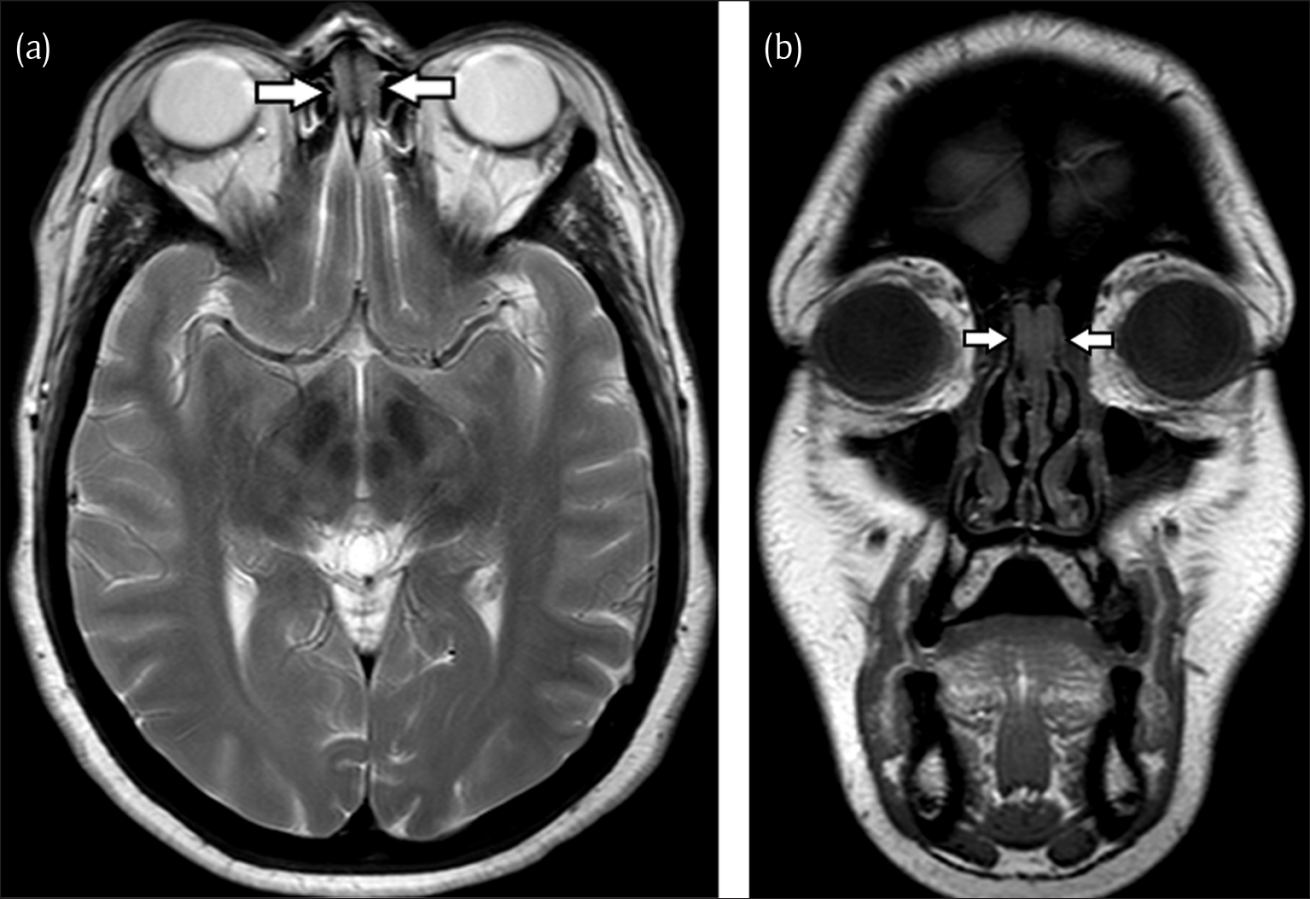
**(a)** On axial T2-weighted images and coronal T1-weighted **(b)** MR images the bilateral mass (arrows) appears iso-intense to white matter. The lesions are sharply delineated and do not involve or cross the cribriform plate.

**Figure 3:**
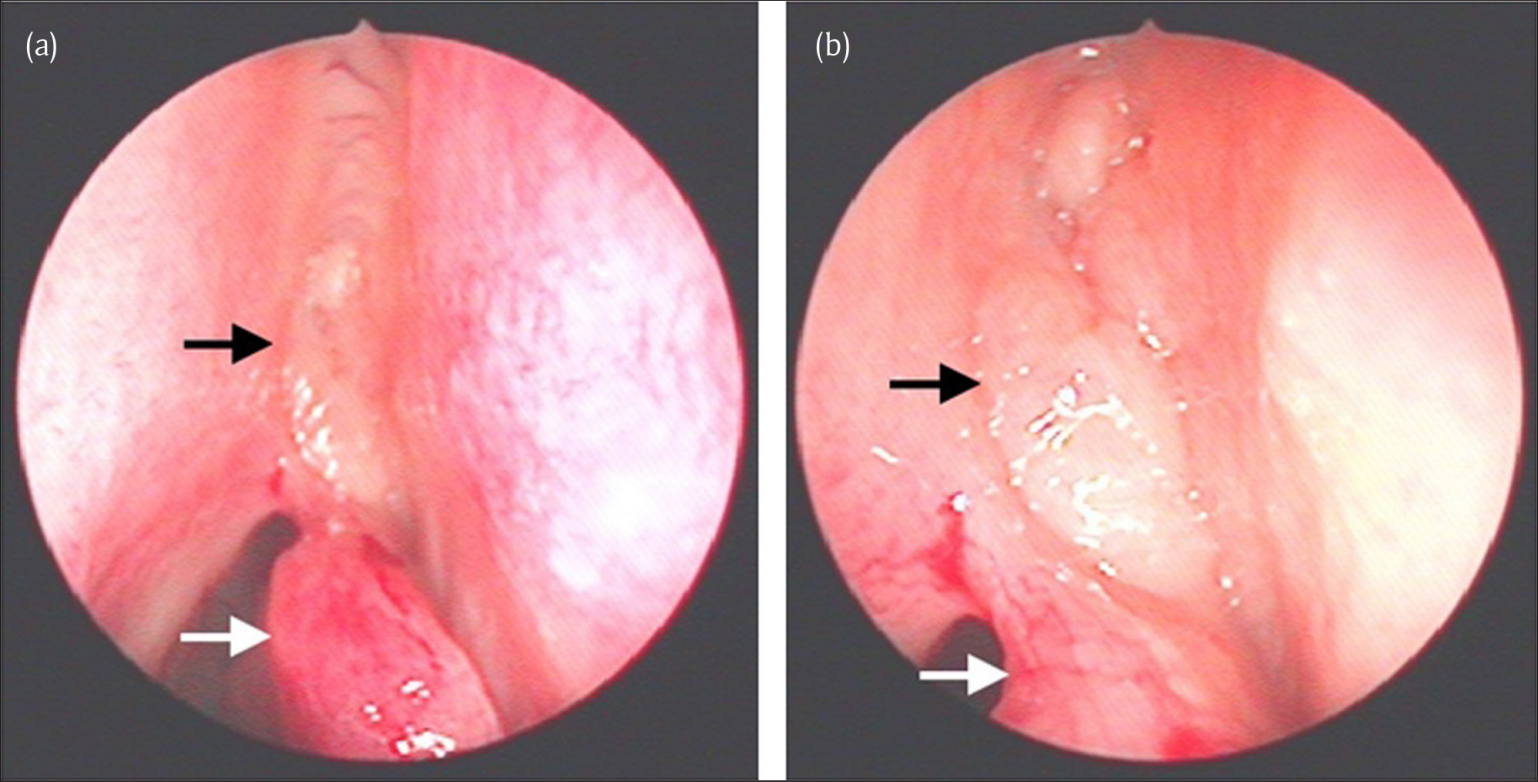
The intra-operative endoscopic images of the right nasal cavity illustrate the gross anatomy of the lesion. A smoothly delineated, oedematous mass (black arrow) is seen cranially in the nose, originating anteriorly and superiorly from the middle concha (white arrow).

A 49-year-old woman presented at the otorhinolaryngology department with symptoms of repeated upper airway infections for six months. She complained of nasal obstruction, headaches, sneezing, hyposmia, postnasal drip and coughing. Treatment with antibiotics and oral steroids had no effect. She had already undergone functional endoscopic sinus surgery with septal correction and partial reduction of a right-sided hypertrophic concha media bullosa in 2008. Endoscopic nasal examination showed a bilateral oedematous mass located medially and cranially in the nose, originating anteriorly of the attachment of the concha media. A computed tomography (CT) (Figure 1) was performed and demonstrated the presence of a bilateral well-delineated soft-tissue mass in the olfactory cleft. There was bone remodelling resulting in widening of the olfactory clefts, but no bone erosion. The mucosa in the paranasal sinuses was only modestly thickened and there was no evidence of sinonasal polyposis. The patient underwent a magnetic resonance (MR) scan for further work-up. On MR (Figure 2) the lesions appeared T1- and T2-isointense compared to white matter. The cribriform plate was intact and there was no intracranial involvement. A biopsy was performed and the presence of REAH was histologically confirmed. Endoscopic non-aggressive resection was performed (Figure 3) and in the follow-up consultation three weeks later the patient was free of symptoms. Nasal endoscopic control four months after surgery showed no signs of recurrence.

## Discussion

Since the lesion was only added to the World Health Organization classification of tumours in 2005 REAH is still a relatively unknown and therefore underdiagnosed entity [2,3]. Thus far it is only sporadically documented in relatively small series and a few case reports.

The lesion is encountered in the third to ninth decade of life and is most commonly diagnosed in the fifth decade. The site of origin is typically the olfactory cleft, which is the area between the superior turbinate, cribriform plate and septum, lined by specialized olfactory epithelium [3]. REAHs can be isolated or associated with nasal polyposis and are present bilaterally in most cases. Patients present with non-specific symptoms of chronic sinonasal inflammatory disease such as nasal obstruction, nasal discharge, facial pain or pressure, headaches and olfactory impairment. Typically, the anosmia does not improve after general steroid treatment. The aetiology and pathogenesis are still unknown, but because of its association with inflammatory disease, several authors suggest that REAH is induced by chronic inflammation. Endoscopically a cerebriform firm pink to yellow mass is observed and histologically the lesion is characterized by a proliferation of the pseudostratified respiratory epithelium, folded into pseudoglands in the underlying interstitial tissue, separated by oedematous or fibrous stroma with mixed chronic inflammatory cell infiltrate. Non-aggressive endoscopic surgical excision is the current treatment of choice. Recurrence rate is reported to be low, but this is still uncertain since only studies with a short follow-up period (up to 3.8 years) are available [3].

The CT imaging findings are described in only a limited number of studies. Lima et al. (51 cases), Hawley et al. (29 cases) and Lee et al. (51 cases) all conclude that REAH causes widening of the olfactory cleft and generally does not cause bone erosion. Hawley et al. states that the sensitivity and specificity for the presence of REAH is 88% and 74% respectively if the width of the olfactory clefts measures 1 cm or more, yielding a positive predictive value of 72% and a negative predictive value of 89%.

To our best knowledge, Braun et al. wrote the only report available on the MRI findings of REAH. In all six patients the lesions reside bilaterally in the olfactory cleft and there is associated nasal polyposis. Their appearance is described as cerebriform, iso-intense to cerebral parenchyma on T1- and T2-weighted images and homogeneously enhancing. The cribriform plate is generally preserved and intracranial extension absent [7].

It is not always possible to distinguish REAH from nasal polyposis. The different predilection site can sometimes aid in the differential diagnosis as REAH originates in the anterior half of the olfactory cleft and nasal polyps in the ethmoidal labyrinth, protruding into the nasal cavity. The effect of oral steroid treatment can also help since sinonasal polyps generally respond to this treatment and REAHs persist [3]. Other important differential diagnoses are adenocarcinomas which generally appear more aggressive and encephaloceles and esthesioneuroblastomas which would be associated with a cribriform plate defect [4,5,6].

## Conclusion

REAH is a relatively new, unknown and underdiagnosed lesion of the nasal cavity, classically originating in the olfactory cleft. On computed tomography and magnetic resonance imaging the diagnosis can be suggested if the olfactory clefts are opacified and the total width is greater than 1 cm. Differentiation from often concurring sinonasal polyposis is difficult clinically as well as on imaging. A trial treatment with oral steroids can be useful as polyps will regress and REAH will persist. Correct identification of this benign entity is important to avoid unnecessarily aggressive surgery.
